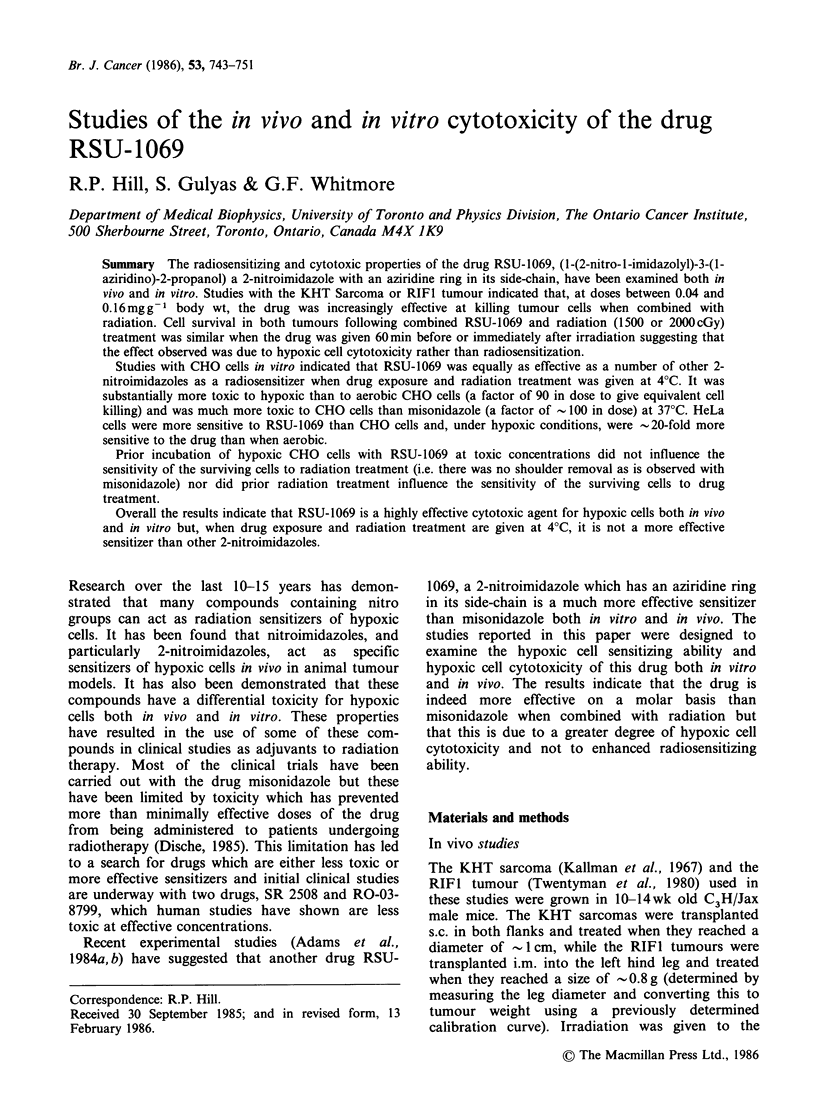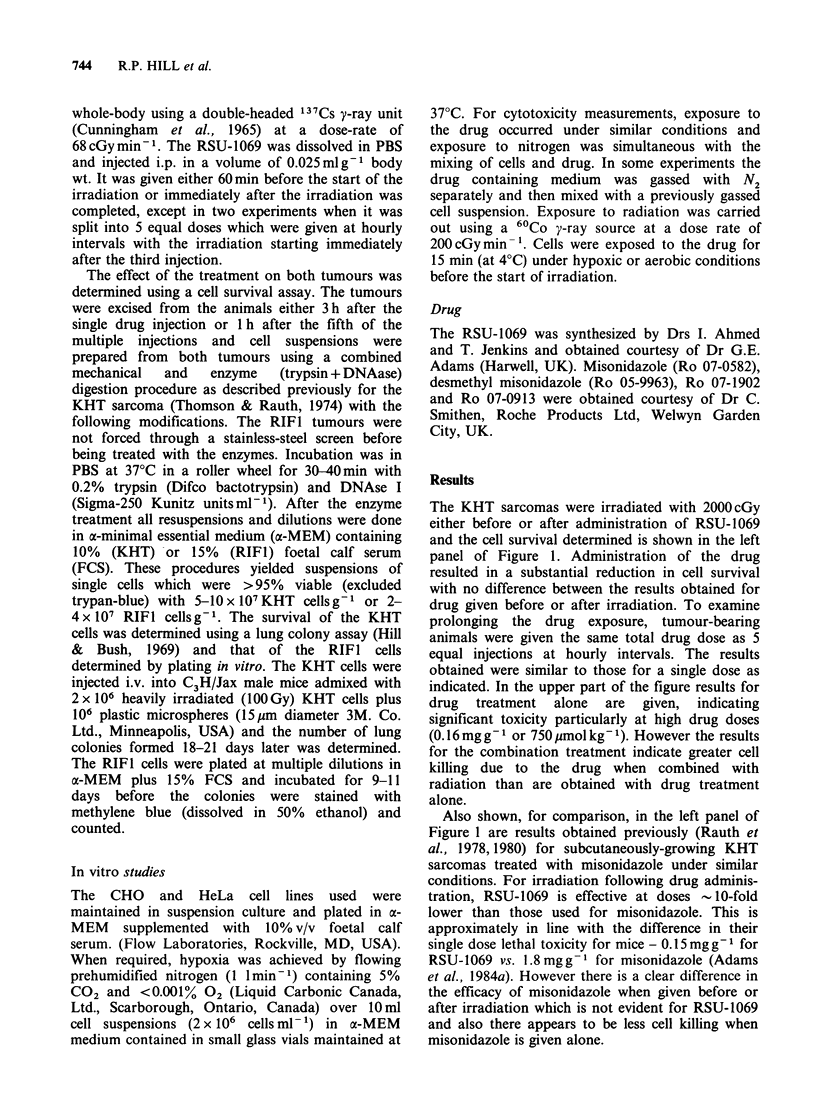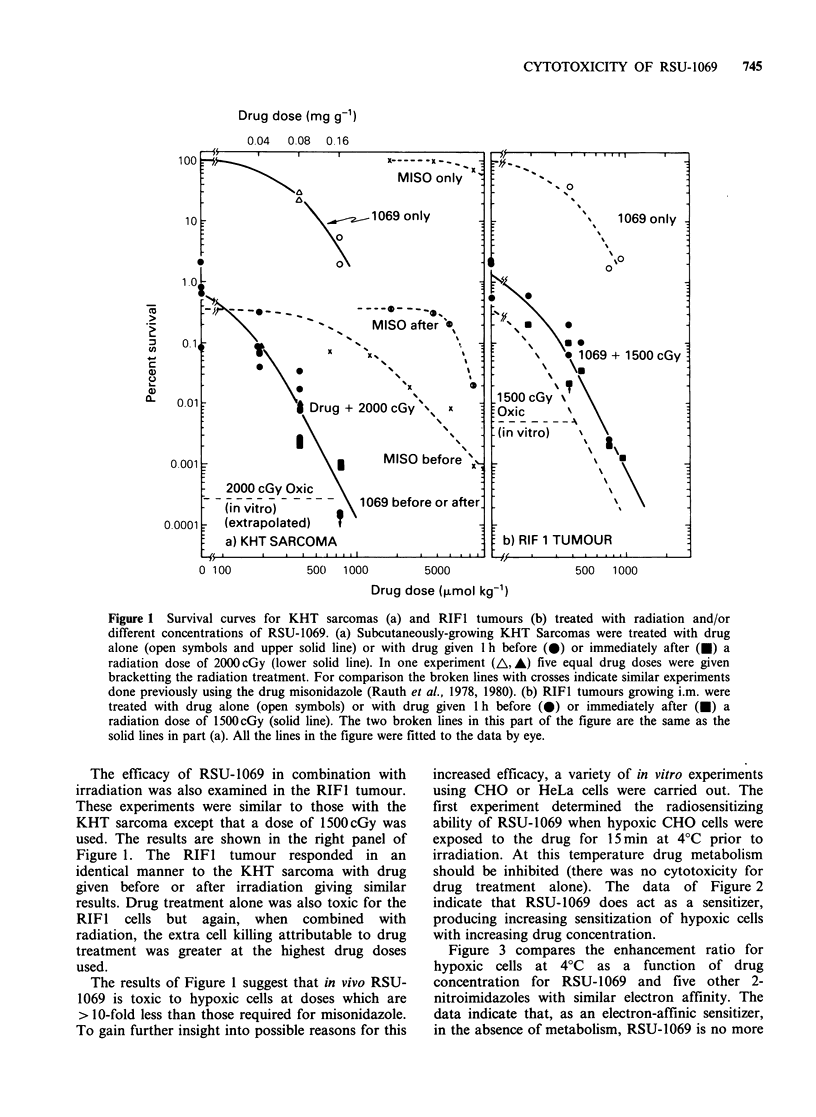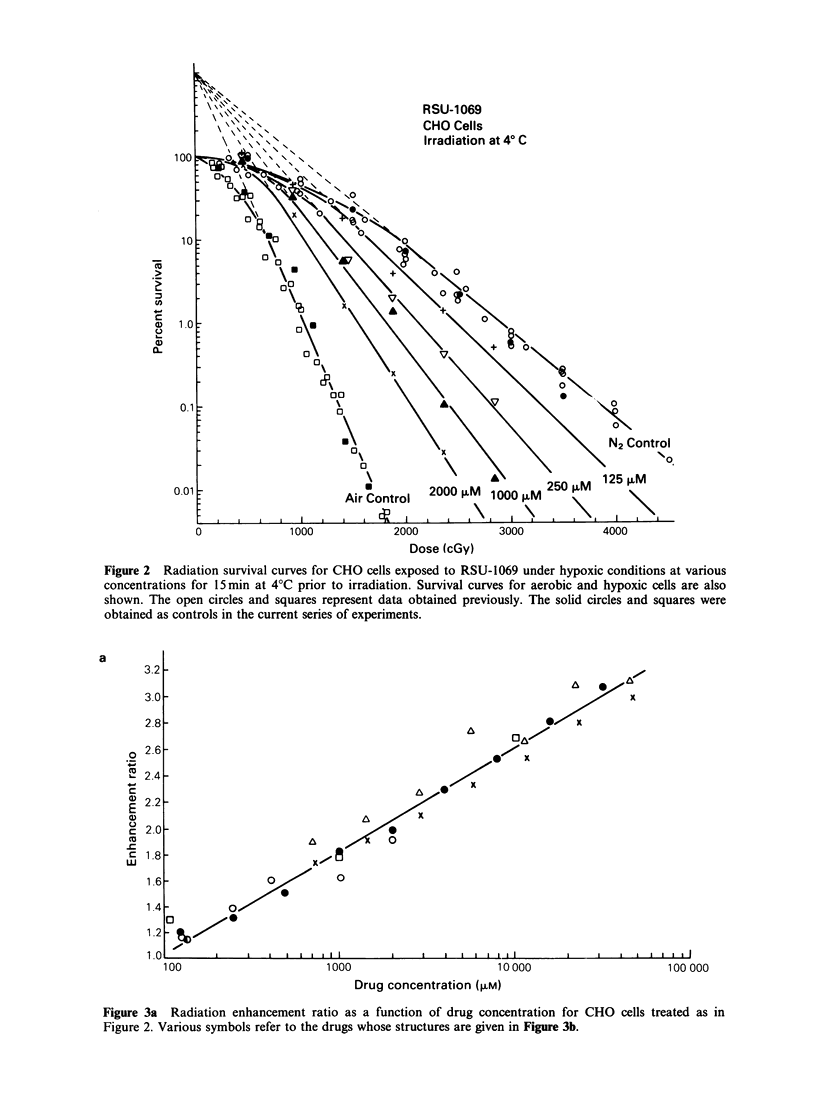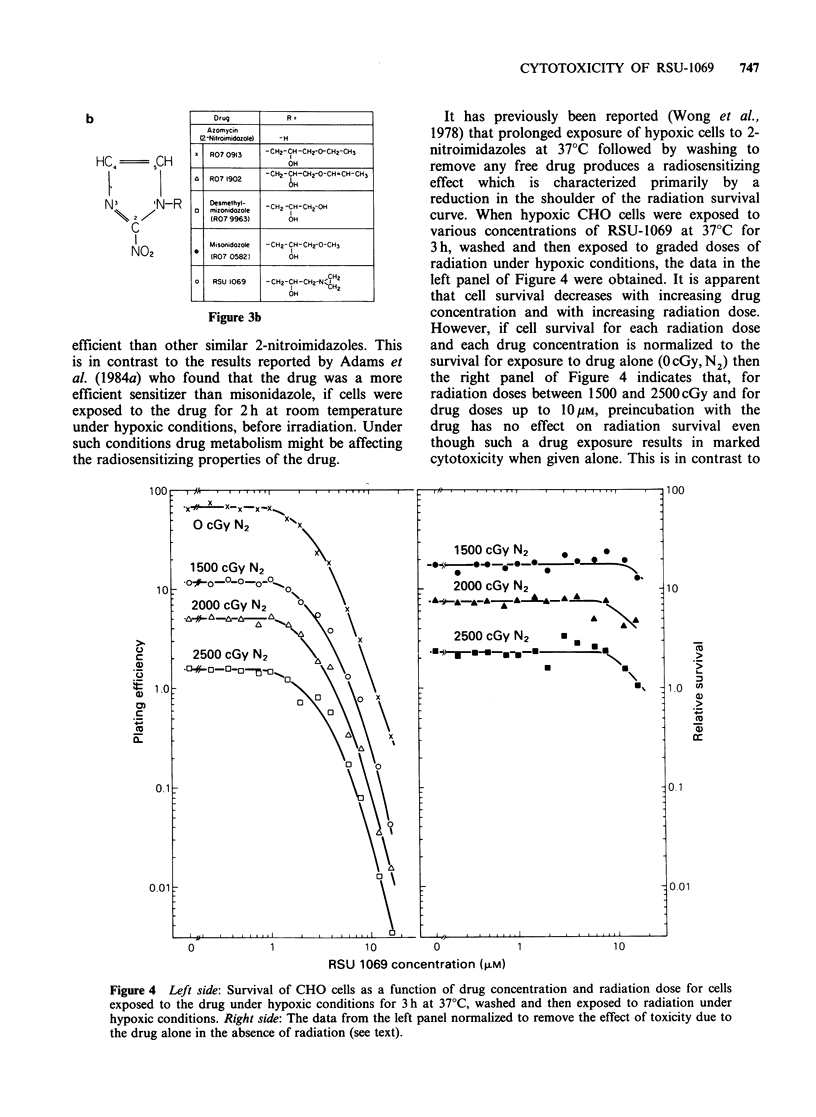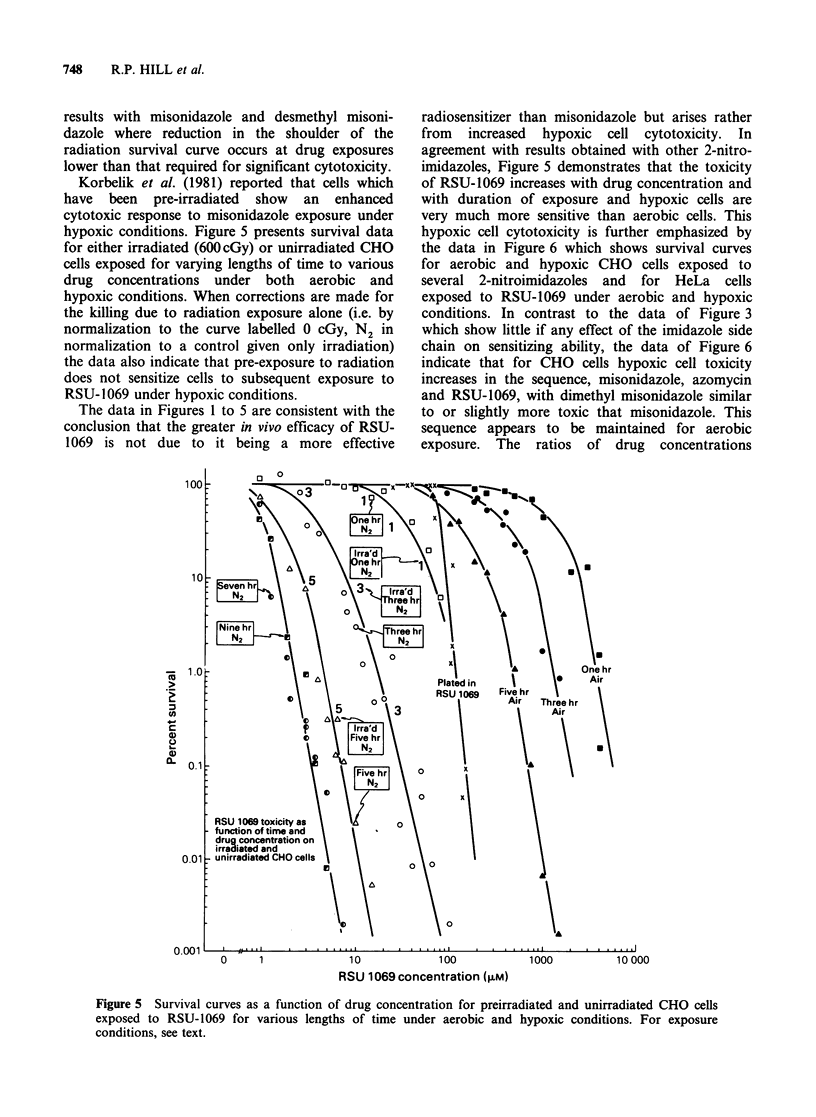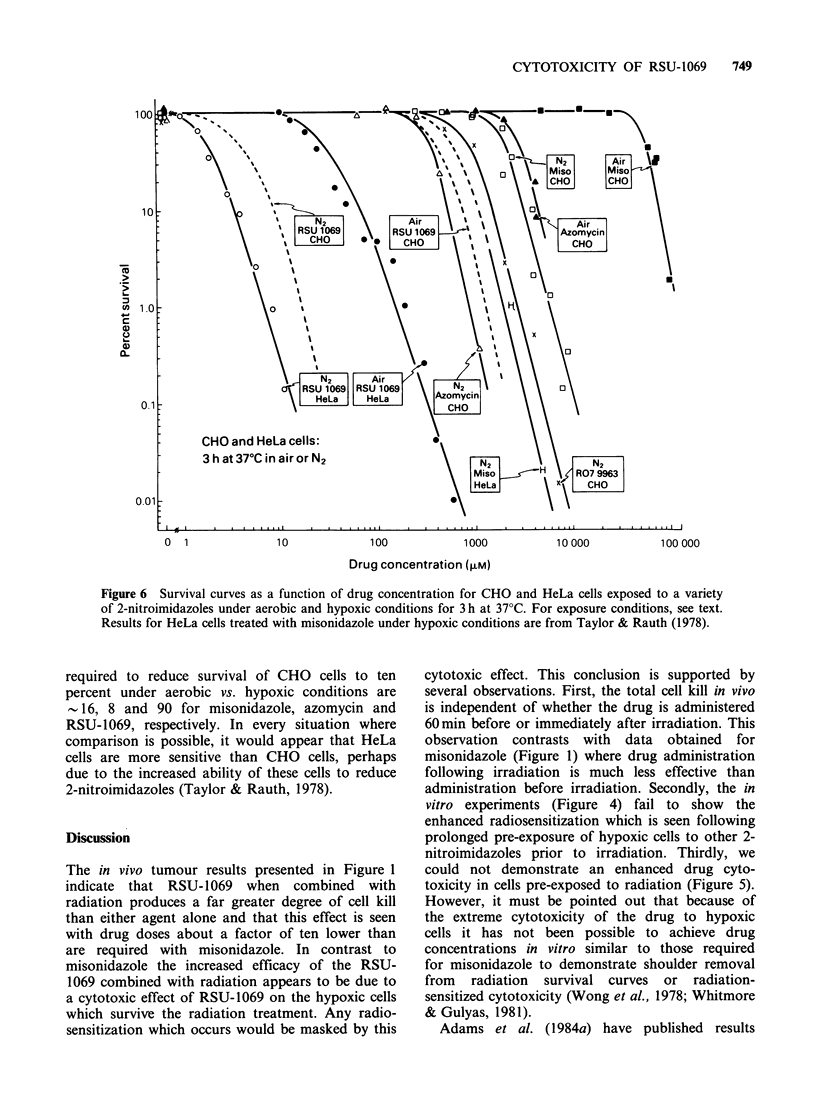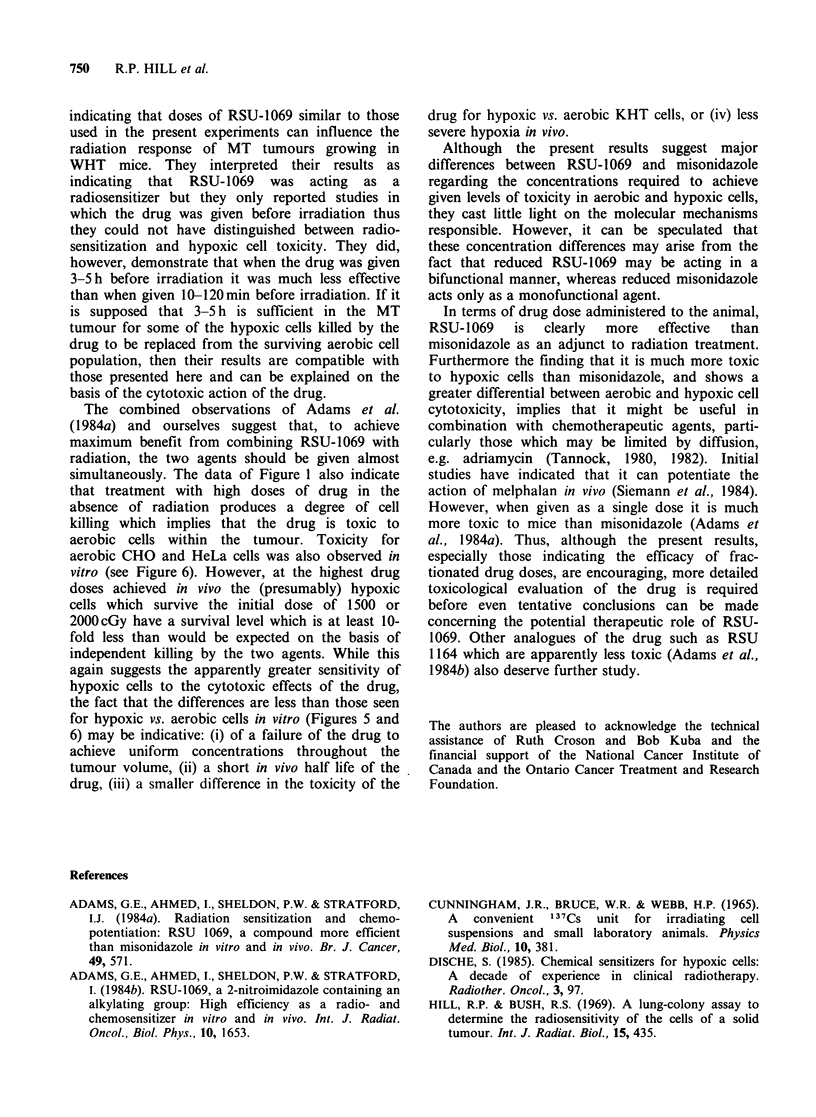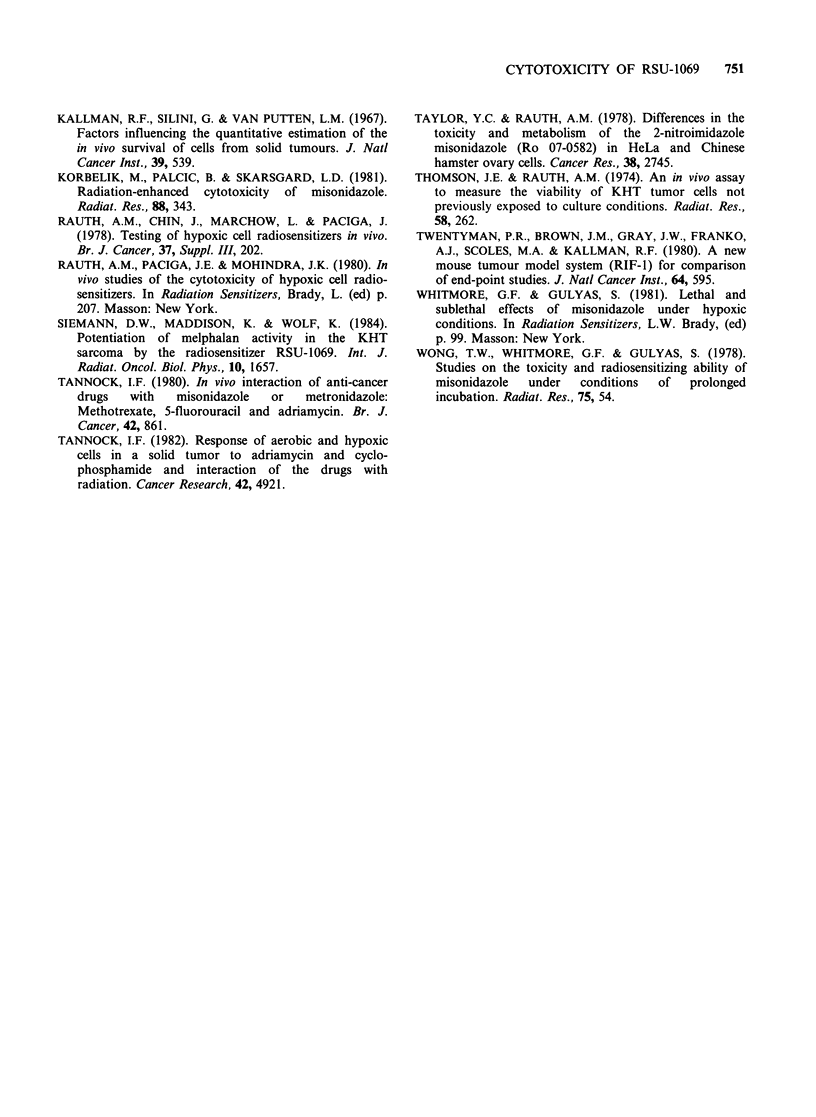# Studies of the in vivo and in vitro cytotoxicity of the drug RSU-1069.

**DOI:** 10.1038/bjc.1986.128

**Published:** 1986-06

**Authors:** R. P. Hill, S. Gulyas, G. F. Whitmore

## Abstract

The radiosensitizing and cytotoxic properties of the drug RSU-1069, (1-(2-nitro-1-imidazolyl)-3-(1-aziridino)-2-propanol) a 2-nitroimidazole with an aziridine ring in its side-chain, have been examined both in vivo and in vitro. Studies with the KHT Sarcoma or RIF1 tumour indicated that, at doses between 0.04 and 0.16 mg g-1 body wt, the drug was increasingly effective at killing tumour cells when combined with radiation. Cell survival in both tumours following combined RSU-1069 and radiation (1500 or 2000 cGy) treatment was similar when the drug was given 60 min before or immediately after irradiation suggesting that the effect observed was due to hypoxic cell cytotoxicity rather than radiosensitization. Studies with CHO cells in vitro indicated that RSU-1069 was equally as effective as a number of other 2-nitroimidazoles as a radiosensitizer when drug exposure and radiation treatment was given at 4 degrees C. It was substantially more toxic to hypoxic than to aerobic CHO cells (a factor of 90 in dose to give equivalent cell killing) and was much more toxic to CHO cells than misonidazole (a factor of approximately 100 in dose) at 37 degrees C. HeLa cells were more sensitive to RSU-1069 than CHO cells and, under hypoxic conditions, were approximately 20-fold more sensitive to the drug than when aerobic. Prior incubation of hypoxic CHO cells with RSU-1069 at toxic concentrations did not influence the sensitivity of the surviving cells to radiation treatment (i.e. there was no shoulder removal as is observed with misonidazole) nor did prior radiation treatment influence the sensitivity of the surviving cells to drug treatment. Overall the results indicate that RSU-1069 is a highly effective cytotoxic agent for hypoxic cells both in vivo and in vitro but, when drug exposure and radiation treatment are given at 4 degrees C, it is not a more effective sensitizer than other 2-nitroimidazoles.


*Br. J. Cancer* (1986), **53**, 743–751

### Studies of the *in vivo* and *in vitro* cytotoxicity of the drug RSU-1069

R.P. Hill, S. Gulyas & G.F. Whitmore

*Department of Medical Biophysics, University of Toronto and Physics Division, The Ontario Cancer Institute, 500 Sherbourne Street, Toronto, Ontario, Canada M4X 1K9*

**Summary** The radiosensitizing and cytotoxic properties of the drug RSU-1069, (1-(2-nitro-1-imidazolyl)-3-(1-aziridino)-2-propanol) a 2-nitroimidazole with an aziridine ring in its side-chain, have been examined both *in vivo* and *in vitro*. Studies with the KHT Sarcoma or RIF1 tumour indicated that, at doses between 0.04 and $0.16\,mg\,g^{-1}$ body wt, the drug was increasingly effective at killing tumour cells when combined with radiation. Cell survival in both tumours following combined RSU-1069 and radiation (1500 or 2000 cGy) treatment was similar when the drug was given 60 min before or immediately after irradiation suggesting that the effect observed was due to hypoxic cell cytotoxicity rather than radiosensitization.

Studies with CHO cells *in vitro* indicated that RSU-1069 was equally as effective as a number of other 2-nitroimidazoles as a radiosensitizer when drug exposure and radiation treatment was given at 4°C. It was substantially more toxic to hypoxic than to aerobic CHO cells (a factor of 90 in dose to give equivalent cell killing) and was much more toxic to CHO cells than misonidazole (a factor of ~100 in dose) at 37°C. HeLa cells were more sensitive to RSU-1069 than CHO cells and, under hypoxic conditions, were ~20-fold more sensitive to the drug than when aerobic.

Prior incubation of hypoxic CHO cells with RSU-1069 at toxic concentrations did not influence the sensitivity of the surviving cells to radiation treatment (i.e. there was no shoulder removal as is observed with misonidazole) nor did prior radiation treatment influence the sensitivity of the surviving cells to drug treatment.

Overall the results indicate that RSU-1069 is a highly effective cytotoxic agent for hypoxic cells both *in vivo* and *in vitro* but, when drug exposure and radiation treatment are given at 4°C, it is not a more effective sensitizer than other 2-nitroimidazoles.

Research over the last 10–15 years has demonstrated that many compounds containing nitro groups can act as radiation sensitizers of hypoxic cells. It has been found that nitroimidazoles, and particularly 2-nitroimidazoles, act as specific sensitizers of hypoxic cells *in vivo* in animal tumour models. It has also been demonstrated that these compounds have a differential toxicity for hypoxic cells both *in vivo* and *in vitro*. These properties have resulted in the use of some of these compounds in clinical studies as adjuvants to radiation therapy. Most of the clinical trials have been carried out with the drug misonidazole but these have been limited by toxicity which has prevented more than minimally effective doses of the drug from being administered to patients undergoing radiotherapy (Dische, 1985). This limitation has led to a search for drugs which are either less toxic or more effective sensitizers and initial clinical studies are underway with two drugs, SR 2508 and RO-03-8799, which human studies have shown are less toxic at effective concentrations.

Recent experimental studies (Adams *et al.*, 1984*a,b*) have suggested that another drug RSU-1069, a 2-nitroimidazole which has an aziridine ring in its side-chain is a much more effective sensitizer than misonidazole both *in vitro* and *in vivo*. The studies reported in this paper were designed to examine the hypoxic cell sensitizing ability and hypoxic cell cytotoxicity of this drug both *in vitro* and *in vivo*. The results indicate that the drug is indeed more effective on a molar basis than misonidazole when combined with radiation but that this is due to a greater degree of hypoxic cell cytotoxicity and not to enhanced radiosensitizing ability.

Correspondence: R.P. Hill.
Received 30 September 1985; and in revised form, 13 February 1986.

### Materials and methods

### In vivo *studies*

The KHT sarcoma (Kallman *et al.*, 1967) and the RIF1 tumour (Twentyman *et al.*, 1980) used in these studies were grown in 10–14 wk old $C_3H$/Jax male mice. The KHT sarcomas were transplanted s.c. in both flanks and treated when they reached a diameter of ~1 cm, while the RIF1 tumours were transplanted i.m. into the left hind leg and treated when they reached a size of ~0.8 g (determined by measuring the leg diameter and converting this to tumour weight using a previously determined calibration curve). Irradiation was given to the

© The Macmillan Press Ltd., 1986

whole-body using a double-headed $^{137}Cs$ γ-ray unit (Cunningham *et al.*, 1965) at a dose-rate of 68 cGy min$^{-1}$. The RSU-1069 was dissolved in PBS and injected i.p. in a volume of 0.025 ml g$^{-1}$ body wt. It was given either 60 min before the start of the irradiation or immediately after the irradiation was completed, except in two experiments when it was split into 5 equal doses which were given at hourly intervals with the irradiation starting immediately after the third injection.

The effect of the treatment on both tumours was determined using a cell survival assay. The tumours were excised from the animals either 3 h after the single drug injection or 1 h after the fifth of the multiple injections and cell suspensions were prepared from both tumours using a combined mechanical and enzyme (trypsin + DNAase) digestion procedure as described previously for the KHT sarcoma (Thomson & Rauth, 1974) with the following modifications. The RIF1 tumours were not forced through a stainless-steel screen before being treated with the enzymes. Incubation was in PBS at 37°C in a roller wheel for 30–40 min with 0.2% trypsin (Difco bactotrypsin) and DNAse I (Sigma-250 Kunitz units ml$^{-1}$). After the enzyme treatment all resuspensions and dilutions were done in α-minimal essential medium (α-MEM) containing 10% (KHT) or 15% (RIF1) foetal calf serum (FCS). These procedures yielded suspensions of single cells which were >95% viable (excluded trypan-blue) with 5–10 × 10$^{7}$ KHT cells g$^{-1}$ or 2–4 × 10$^{7}$ RIF1 cells g$^{-1}$. The survival of the KHT cells was determined using a lung colony assay (Hill & Bush, 1969) and that of the RIF1 cells determined by plating *in vitro*. The KHT cells were injected i.v. into $C_3H$/Jax male mice admixed with 2 × 10$^{6}$ heavily irradiated (100 Gy) KHT cells plus 10$^{6}$ plastic microspheres (15 μm diameter 3M. Co. Ltd., Minneapolis, USA) and the number of lung colonies formed 18–21 days later was determined. The RIF1 cells were plated at multiple dilutions in α-MEM plus 15% FCS and incubated for 9–11 days before the colonies were stained with methylene blue (dissolved in 50% ethanol) and counted.

### In vitro *studies*

The CHO and HeLa cell lines used were maintained in suspension culture and plated in α-MEM supplemented with 10% v/v foetal calf serum. (Flow Laboratories, Rockville, MD, USA). When required, hypoxia was achieved by flowing prehumidified nitrogen (1 l min$^{-1}$) containing 5% $CO_2$ and <0.001% $O_2$ (Liquid Carbonic Canada, Ltd., Scarborough, Ontario, Canada) over 10 ml cell suspensions (2 × 10$^{6}$ cells ml$^{-1}$) in α-MEM medium contained in small glass vials maintained at 37°C. For cytotoxicity measurements, exposure to the drug occurred under similar conditions and exposure to nitrogen was simultaneous with the mixing of cells and drug. In some experiments the drug containing medium was gassed with $N_2$ separately and then mixed with a previously gassed cell suspension. Exposure to radiation was carried out using a $^{60}Co$ γ-ray source at a dose rate of 200 cGy min$^{-1}$. Cells were exposed to the drug for 15 min (at 4°C) under hypoxic or aerobic conditions before the start of irradiation.

### *Drug*

The RSU-1069 was synthesized by Drs I. Ahmed and T. Jenkins and obtained courtesy of Dr G.E. Adams (Harwell, UK). Misonidazole (Ro 07-0582), desmethyl misonidazole (Ro 05-9963), Ro 07-1902 and Ro 07-0913 were obtained courtesy of Dr C. Smithen, Roche Products Ltd, Welwyn Garden City, UK.

### Results

The KHT sarcomas were irradiated with 2000 cGy either before or after administration of RSU-1069 and the cell survival determined is shown in the left panel of Figure 1. Administration of the drug resulted in a substantial reduction in cell survival with no difference between the results obtained for drug given before or after irradiation. To examine prolonging the drug exposure, tumour-bearing animals were given the same total drug dose as 5 equal injections at hourly intervals. The results obtained were similar to those for a single dose as indicated. In the upper part of the figure results for drug treatment alone are given, indicating significant toxicity particularly at high drug doses (0.16 mg g$^{-1}$ or 750 μmol kg$^{-1}$). However the results for the combination treatment indicate greater cell killing due to the drug when combined with radiation than are obtained with drug treatment alone.

Also shown, for comparison, in the left panel of Figure 1 are results obtained previously (Rauth *et al.*, 1978, 1980) for subcutaneously-growing KHT sarcomas treated with misonidazole under similar conditions. For irradiation following drug administration, RSU-1069 is effective at doses ~10-fold lower than those used for misonidazole. This is approximately in line with the difference in their single dose lethal toxicity for mice – 0.15 mg g$^{-1}$ for RSU-1069 *vs.* 1.8 mg g$^{-1}$ for misonidazole (Adams *et al.*, 1984*a*). However there is a clear difference in the efficacy of misonidazole when given before or after irradiation which is not evident for RSU-1069 and also there appears to be less cell killing when misonidazole is given alone.

**Figure 1** Survival curves for KHT sarcomas (a) and RIF1 tumours (b) treated with radiation and/or different concentrations of RSU-1069. (a) Subcutaneously-growing KHT Sarcomas were treated with drug alone (open symbols and upper solid line) or with drug given 1 h before (●) or immediately after (■) a radiation dose of 2000 cGy (lower solid line). In one experiment (△, ▲) five equal drug doses were given bracketting the radiation treatment. For comparison the broken lines with crosses indicate similar experiments done previously using the drug misonidazole (Rauth *et al.*, 1978, 1980). (b) RIF1 tumours growing i.m. were treated with drug alone (open symbols) or with drug given 1 h before (●) or immediately after (■) a radiation dose of 1500 cGy (solid line). The two broken lines in this part of the figure are the same as the solid lines in part (a). All the lines in the figure were fitted to the data by eye.

The efficacy of RSU-1069 in combination with irradiation was also examined in the RIF1 tumour. These experiments were similar to those with the KHT sarcoma except that a dose of 1500 cGy was used. The results are shown in the right panel of Figure 1. The RIF1 tumour responded in an identical manner to the KHT sarcoma with drug given before or after irradiation giving similar results. Drug treatment alone was also toxic for the RIF1 cells but again, when combined with radiation, the extra cell killing attributable to drug treatment was greater at the highest drug doses used.

The results of Figure 1 suggest that *in vivo* RSU-1069 is toxic to hypoxic cells at doses which are >10-fold less than those required for misonidazole. To gain further insight into possible reasons for this increased efficacy, a variety of *in vitro* experiments using CHO or HeLa cells were carried out. The first experiment determined the radiosensitizing ability of RSU-1069 when hypoxic CHO cells were exposed to the drug for 15 min at 4°C prior to irradiation. At this temperature drug metabolism should be inhibited (there was no cytotoxicity for drug treatment alone). The data of Figure 2 indicate that RSU-1069 does act as a sensitizer, producing increasing sensitization of hypoxic cells with increasing drug concentration.

Figure 3 compares the enhancement ratio for hypoxic cells at 4°C as a function of drug concentration for RSU-1069 and five other 2-nitroimidazoles with similar electron affinity. The data indicate that, as an electron-affinic sensitizer, in the absence of metabolism, RSU-1069 is no more

**Figure 2** Radiation survival curves for CHO cells exposed to RSU-1069 under hypoxic conditions at various concentrations for 15 min at 4°C prior to irradiation. Survival curves for aerobic and hypoxic cells are also shown. The open circles and squares represent data obtained previously. The solid circles and squares were obtained as controls in the current series of experiments.

**Figure 3a** Radiation enhancement ratio as a function of drug concentration for CHO cells treated as in Figure 2. Various symbols refer to the drugs whose structures are given in **Figure 3b**.

**Figure 3b**

efficient than other similar 2-nitroimidazoles. This is in contrast to the results reported by Adams *et al.* (1984*a*) who found that the drug was a more efficient sensitizer than misonidazole, if cells were exposed to the drug for 2 h at room temperature under hypoxic conditions, before irradiation. Under such conditions drug metabolism might be affecting the radiosensitizing properties of the drug.

It has previously been reported (Wong *et al.*, 1978) that prolonged exposure of hypoxic cells to 2-nitroimidazoles at 37°C followed by washing to remove any free drug produces a radiosensitizing effect which is characterized primarily by a reduction in the shoulder of the radiation survival curve. When hypoxic CHO cells were exposed to various concentrations of RSU-1069 at 37°C for 3 h, washed and then exposed to graded doses of radiation under hypoxic conditions, the data in the left panel of Figure 4 were obtained. It is apparent that cell survival decreases with increasing drug concentration and with increasing radiation dose. However, if cell survival for each radiation dose and each drug concentration is normalized to the survival for exposure to drug alone (0 cGy, $N_2$) then the right panel of Figure 4 indicates that, for radiation doses between 1500 and 2500 cGy and for drug doses up to 10 $\mu$M, preincubation with the drug has no effect on radiation survival even though such a drug exposure results in marked cytotoxicity when given alone. This is in contrast to

**Figure 4** *Left side*: Survival of CHO cells as a function of drug concentration and radiation dose for cells exposed to the drug under hypoxic conditions for 3 h at 37°C, washed and then exposed to radiation under hypoxic conditions. *Right side*: The data from the left panel normalized to remove the effect of toxicity due to the drug alone in the absence of radiation (see text).

results with misonidazole and desmethyl misonidazole where reduction in the shoulder of the radiation survival curve occurs at drug exposures lower than that required for significant cytotoxicity.

Korbelik *et al.* (1981) reported that cells which have been pre-irradiated show an enhanced cytotoxic response to misonidazole exposure under hypoxic conditions. Figure 5 presents survival data for either irradiated (600 cGy) or unirradiated CHO cells exposed for varying lengths of time to various drug concentrations under both aerobic and hypoxic conditions. When corrections are made for the killing due to radiation exposure alone (i.e. by normalization to the curve labelled 0 cGy, $N_2$ in normalization to a control given only irradiation) the data also indicate that pre-exposure to radiation does not sensitize cells to subsequent exposure to RSU-1069 under hypoxic conditions.

The data in Figures 1 to 5 are consistent with the conclusion that the greater *in vivo* efficacy of RSU-1069 is not due to it being a more effective radiosensitizer than misonidazole but arises rather from increased hypoxic cell cytotoxicity. In agreement with results obtained with other 2-nitroimidazoles, Figure 5 demonstrates that the toxicity of RSU-1069 increases with drug concentration and with duration of exposure and hypoxic cells are very much more sensitive than aerobic cells. This hypoxic cell cytotoxicity is further emphasized by the data in Figure 6 which shows survival curves for aerobic and hypoxic CHO cells exposed to several 2-nitroimidazoles and for HeLa cells exposed to RSU-1069 under aerobic and hypoxic conditions. In contrast to the data of Figure 3 which show little if any effect of the imidazole side chain on sensitizing ability, the data of Figure 6 indicate that for CHO cells hypoxic cell toxicity increases in the sequence, misonidazole, azomycin and RSU-1069, with dimethyl misonidazole similar to or slightly more toxic that misonidazole. This sequence appears to be maintained for aerobic exposure. The ratios of drug concentrations

**Figure 5** Survival curves as a function of drug concentration for preirradiated and unirradiated CHO cells exposed to RSU-1069 for various lengths of time under aerobic and hypoxic conditions. For exposure conditions, see text.

**Figure 6** Survival curves as a function of drug concentration for CHO and HeLa cells exposed to a variety of 2-nitroimidazoles under aerobic and hypoxic conditions for 3 h at 37°C. For exposure conditions, see text. Results for HeLa cells treated with misonidazole under hypoxic conditions are from Taylor & Rauth (1978).

required to reduce survival of CHO cells to ten percent under aerobic *vs.* hypoxic conditions are ~16, 8 and 90 for misonidazole, azomycin and RSU-1069, respectively. In every situation where comparison is possible, it would appear that HeLa cells are more sensitive than CHO cells, perhaps due to the increased ability of these cells to reduce 2-nitroimidazoles (Taylor & Rauth, 1978).

### Discussion

The *in vivo* tumour results presented in Figure 1 indicate that RSU-1069 when combined with radiation produces a far greater degree of cell kill than either agent alone and that this effect is seen with drug doses about a factor of ten lower than are required with misonidazole. In contrast to misonidazole the increased efficacy of the RSU-1069 combined with radiation appears to be due to a cytotoxic effect of RSU-1069 on the hypoxic cells which survive the radiation treatment. Any radiosensitization which occurs would be masked by this cytotoxic effect. This conclusion is supported by several observations. First, the total cell kill *in vivo* is independent of whether the drug is administered 60 min before or immediately after irradiation. This observation contrasts with data obtained for misonidazole (Figure 1) where drug administration following irradiation is much less effective than administration before irradiation. Secondly, the *in vitro* experiments (Figure 4) fail to show the enhanced radiosensitization which is seen following prolonged pre-exposure of hypoxic cells to other 2-nitroimidazoles prior to irradiation. Thirdly, we could not demonstrate an enhanced drug cytotoxicity in cells pre-exposed to radiation (Figure 5). However, it must be pointed out that because of the extreme cytotoxicity of the drug to hypoxic cells it has not been possible to achieve drug concentrations *in vitro* similar to those required for misonidazole to demonstrate shoulder removal from radiation survival curves or radiation-sensitized cytotoxicity (Wong *et al.*, 1978; Whitmore & Gulyas, 1981).

Adams *et al.* (1984*a*) have published results indicating that doses of RSU-1069 similar to those used in the present experiments can influence the radiation response of MT tumours growing in WHT mice. They interpreted their results as indicating that RSU-1069 was acting as a radiosensitizer but they only reported studies in which the drug was given before irradiation thus they could not have distinguished between radiosensitization and hypoxic cell toxicity. They did, however, demonstrate that when the drug was given 3–5 h before irradiation it was much less effective than when given 10–120 min before irradiation. If it is supposed that 3–5 h is sufficient in the MT tumour for some of the hypoxic cells killed by the drug to be replaced from the surviving aerobic cell population, then their results are compatible with those presented here and can be explained on the basis of the cytotoxic action of the drug.

The combined observations of Adams *et al.* (1984*a*) and ourselves suggest that, to achieve maximum benefit from combining RSU-1069 with radiation, the two agents should be given almost simultaneously. The data of Figure 1 also indicate that treatment with high doses of drug in the absence of radiation produces a degree of cell killing which implies that the drug is toxic to aerobic cells within the tumour. Toxicity for aerobic CHO and HeLa cells was also observed *in vitro* (see Figure 6). However, at the highest drug doses achieved *in vivo* the (presumably) hypoxic cells which survive the initial dose of 1500 or 2000 cGy have a survival level which is at least 10-fold less than would be expected on the basis of independent killing by the two agents. While this again suggests the apparently greater sensitivity of hypoxic cells to the cytotoxic effects of the drug, the fact that the differences are less than those seen for hypoxic *vs.* aerobic cells *in vitro* (Figures 5 and 6) may be indicative: (i) of a failure of the drug to achieve uniform concentrations throughout the tumour volume, (ii) a short *in vivo* half life of the drug, (iii) a smaller difference in the toxicity of the drug for hypoxic *vs.* aerobic KHT cells, or (iv) less severe hypoxia *in vivo*.

Although the present results suggest major differences between RSU-1069 and misonidazole regarding the concentrations required to achieve given levels of toxicity in aerobic and hypoxic cells, they cast little light on the molecular mechanisms responsible. However, it can be speculated that these concentration differences may arise from the fact that reduced RSU-1069 may be acting in a bifunctional manner, whereas reduced misonidazole acts only as a monofunctional agent.

In terms of drug dose administered to the animal, RSU-1069 is clearly more effective than misonidazole as an adjunct to radiation treatment. Furthermore the finding that it is much more toxic to hypoxic cells than misonidazole, and shows a greater differential between aerobic and hypoxic cell cytotoxicity, implies that it might be useful in combination with chemotherapeutic agents, particularly those which may be limited by diffusion, e.g. adriamycin (Tannock, 1980, 1982). Initial studies have indicated that it can potentiate the action of melphalan *in vivo* (Siemann *et al.*, 1984). However, when given as a single dose it is much more toxic to mice than misonidazole (Adams *et al.*, 1984*a*). Thus, although the present results, especially those indicating the efficacy of fractionated drug doses, are encouraging, more detailed toxicological evaluation of the drug is required before even tentative conclusions can be made concerning the potential therapeutic role of RSU-1069. Other analogues of the drug such as RSU 1164 which are apparently less toxic (Adams *et al.*, 1984*b*) also deserve further study.

The authors are pleased to acknowledge the technical assistance of Ruth Croson and Bob Kuba and the financial support of the National Cancer Institute of Canada and the Ontario Cancer Treatment and Research Foundation.

### References

ADAMS, G.E., AHMED, I., SHELDON, P.W. & STRATFORD, I.J. (1984*a*). Radiation sensitization and chemopotentiation: RSU 1069, a compound more efficient than misonidazole *in vitro* and *in vivo*. *Br. J. Cancer*, **49**, 571.

ADAMS, G.E., AHMED, I., SHELDON, P.W. & STRATFORD, I. (1984*b*). RSU-1069, a 2-nitroimidazole containing an alkylating group: High efficiency as a radio- and chemosensitizer *in vitro* and *in vivo*. *Int. J. Radiat. Oncol., Biol. Phys.*, **10**, 1653.

CUNNINGHAM, J.R., BRUCE, W.R. & WEBB, H.P. (1965). A convenient $^{137}Cs$ unit for irradiating cell suspensions and small laboratory animals. *Physics Med. Biol.*, **10**, 381.

DISCHE, S. (1985). Chemical sensitizers for hypoxic cells: A decade of experience in clinical radiotherapy. *Radiother. Oncol.*, **3**, 97.

HILL, R.P. & BUSH, R.S. (1969). A lung-colony assay to determine the radiosensitivity of the cells of a solid tumour. *Int. J. Radiat. Biol.*, **15**, 435.

KALLMAN, R.F., SILINI, G. & VAN PUTTEN, L.M. (1967). Factors influencing the quantitative estimation of the *in vivo* survival of cells from solid tumours. *J. Natl Cancer Inst.*, **39**, 539.

KORBELIK, M., PALCIC, B. & SKARSGARD, L.D. (1981). Radiation-enhanced cytotoxicity of misonidazole. *Radiat. Res.*, **88**, 343.

RAUTH, A.M., CHIN, J., MARCHOW, L. & PACIGA, J. (1978). Testing of hypoxic cell radiosensitizers *in vivo*. *Br. J. Cancer*, **37**, *Suppl. III*, 202.

RAUTH, A.M., PACIGA, J.E. & MOHINDRA, J.K. (1980). *In vivo* studies of the cytotoxicity of hypoxic cell radiosensitizers. In *Radiation Sensitizers*, Brady, L. (ed) p. 207. Masson: New York.

SIEMANN, D.W., MADDISON, K. & WOLF, K. (1984). Potentiation of melphalan activity in the KHT sarcoma by the radiosensitizer RSU-1069. *Int. J. Radiat. Oncol. Biol. Phys.*, **10**, 1657.

TANNOCK, I.F. (1980). *In vivo* interaction of anti-cancer drugs with misonidazole or metronidazole: Methotrexate, 5-fluorouracil and adriamycin. *Br. J. Cancer*, **42**, 861.

TANNOCK, I.F. (1982). Response of aerobic and hypoxic cells in a solid tumor to adriamycin and cyclophosphamide and interaction of the drugs with radiation. *Cancer Research*, **42**, 4921.

TAYLOR, Y.C. & RAUTH, A.M. (1978). Differences in the toxicity and metabolism of the 2-nitroimidazole misonidazole (Ro 07-0582) in HeLa and Chinese hamster ovary cells. *Cancer Res.*, **38**, 2745.

THOMSON, J.E. & RAUTH, A.M. (1974). An *in vivo* assay to measure the viability of KHT tumor cells not previously exposed to culture conditions. *Radiat. Res.*, **58**, 262.

TWENTYMAN, P.R., BROWN, J.M., GRAY, J.W., FRANKO, A.J., SCOLES, M.A. & KALLMAN, R.F. (1980). A new mouse tumour model system (RIF-1) for comparison of end-point studies. *J. Natl Cancer Inst.*, **64**, 595.

WHITMORE, G.F. & GULYAS, S. (1981). Lethal and sublethal effects of misonidazole under hypoxic conditions. In *Radiation Sensitizers*, L.W. Brady, (ed) p. 99. Masson: New York.

WONG, T.W., WHITMORE, G.F. & GULYAS, S. (1978). Studies on the toxicity and radiosensitizing ability of misonidazole under conditions of prolonged incubation. *Radiat. Res.*, **75**, 54.